# Yeast Gcn2 retains activity following humanization of its auto‐phosphorylation region

**DOI:** 10.1002/2211-5463.70286

**Published:** 2026-08-02

**Authors:** Reuben A. Anderson, Anja H. Schiemann, Evelyn Sattlegger

**Affiliations:** ^1^ School of Food Technology and Natural Sciences Massey University Palmerston North New Zealand; ^2^ Maurice Wilkins Centre for Molecular BioDiscovery Palmerston North New Zealand

**Keywords:** auto‐phosphorylation, Gcn1, Gcn2, ISR, protein kinase, stress response

## Abstract

The protein kinase Gcn2 is a conserved component of a eukaryotic signaling pathway best known for helping cells cope with amino acid shortage. Upon starvation, Gcn2 auto‐phosphorylates and then phosphorylates eIF2*α*, triggering widespread changes in gene expression. While Gcn2 is gaining attention for its diverse biological roles and links to various diseases, its activation and regulation remain unclear. To date, *Saccharomyces cerevisiae* remains an important model for dissecting these mechanisms in detail. However, commercial antibodies recognizing phosphorylated Gcn2 are available only for mammalian GCN2. Therefore, we engineered a yeast Gcn2 variant, Gcn2‐HsC, recognizable by these antibodies. Gcn2‐HsC almost completely complemented a *gcn2Δ* strain, retained its ability to phosphorylate eIF2*α*, and is still dependent on Gcn1 for function. Ultimately, our results suggest that Gcn2‐HsC serves as a valuable tool for Gcn2‐related studies in the highly tractable yeast system.

## Graphical abstract

Using *Saccharomyces cerevisiae* as a model to study Gcn2 activation and regulation is limited by the lack of antibodies detecting phosphorylated Gcn2. To overcome this, we engineered Gcn2‐HsC, a yeast Gcn2 variant recognizable by commercial anti‐human phospho‐GCN2 antibodies. Gcn2‐HsC retained near‐normal function, phosphorylated eIF2*α* efficiently, and remained dependent on Gcn1, providing a new tool to directly study yeast Gcn2 phosphorylation.

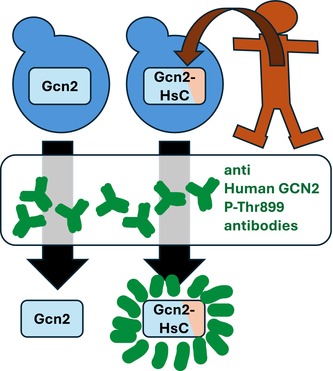

**Abbreviations**
3AT: 3‐amino‐1,2,4‐triazole
eIF2*α*
: *α* subunit of eukaryotic translation initiation factor 2
eIF2*α*‐P: phosphorylated eIF2*α*
Gcn: General control nonderepressible
Gcn2‐P: auto‐phosphorylated Gcn2
HsGCN2: *Homo sapiens* GCN2
ScGcn2: *Saccharomyces cerevisiae* Gcn2

Gcn2 is a protein kinase integral to a signal transduction pathway that is conserved across the eukaryotic kingdom [1, 2, 3]. It is primarily recognized for its critical role in enabling cells to survive amino acid starvation by initiating a stress response. When cells experience amino acid shortage, Gcn2 undergoes auto‐phosphorylation to then phosphorylate eIF2*α*, which results in a shift in the cell's gene expression profile, including the activation of genes involved in stress response and metabolic adaptation.

Gcn2 is attracting increasing interest due to its involvement in a broad spectrum of biological processes. For instance, GCN2 contributes to cellular adaptation under conditions, such as purine or pyrimidine limitation, glucose deprivation, oxidative stress, and UV exposure. In higher eukaryotes, its functions have expanded to include roles in antiviral defense, regulation of feeding behavior, long‐term memory formation, neuronal development, and immune responses. Additionally, GCN2 has been linked to various pathological conditions, including neurodegenerative diseases, cancer, inflammatory disorders, and metabolic syndromes [3, 4, 5]. GCN2 deletion in mice is not lethal [6], making GCN2 an attractive drug target for treating GCN2‐associated diseases and disorders [7]. Despite its importance, the precise mechanisms underlying GCN2 activation and regulation remain poorly understood. Hence, filling this knowledge gap is particularly important to support efforts in seeking effective GCN2 inhibitors, and clarifying their modes of action to guide rational drug development and optimization. While mammalian systems are used to understand molecular mechanisms surrounding GCN2, *Saccharomyces cerevisiae* (baker's yeast) still serves as an excellent model system for unraveling these mechanisms in minute detail, thanks to its genetic tractability and amenability to a wide range of experimental techniques [8, 9, 10, 11, 12]. However, a limitation in studying Gcn2 in yeast has been the lack of suitable antibodies that can detect the phosphorylated form of Gcn2 (Gcn2‐P). Antibodies had been raised against auto‐phosphorylated yeast Gcn2 previously; however, the detection of *in vivo* Gcn2‐P levels required prior immunoprecipitation [13]. Commercially available anti Gcn2‐P specific antibodies only detect mammalian GCN2.

In this study, we aimed to modify yeast Gcn2 such that its auto‐phosphorylation can be easily detected using commercially available antibodies. In *Saccharomyces cerevisiae* Gcn2 (ScGcn2), key auto‐phosphorylation sites are Thr‐882 and Thr‐887 in the protein kinase activation loop, with Thr‐887 being the most critical one [14]. In mammals, the corresponding auto‐phosphorylation sites are found in analogous regions, and are Thr‐898 and Thr‐903 in mouse, and Thr‐899 and Thr‐904 in human, with Thr‐898/899 being equivalent to yeast Thr‐887 [14, 15]. The surrounding sequences of these phosphorylation sites in ScGcn2 and human GCN2 (HsGCN2) are moderately conserved, reflecting their shared evolutionary origin and similar functional roles (Fig. 1A).

**Fig. 1:**
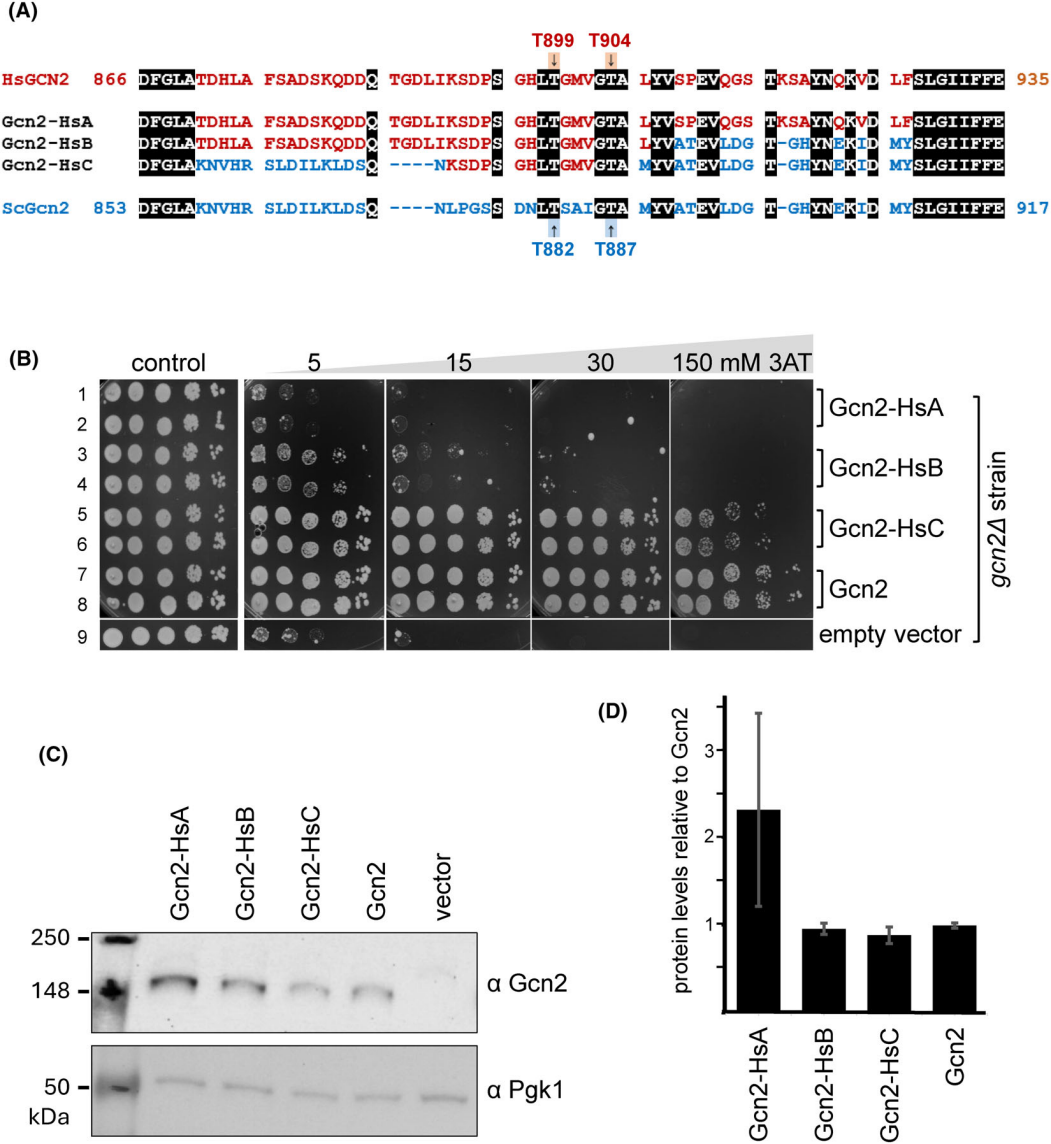
Humanized ScGcn2 variant Gcn2‐HsC almost completely complements the 3AT^s^ phenotype of a *gcn2Δ* strain. (A) Multiple sequence alignment of *Homo sapiens* GCN2 (HsGCN2, Accession: NP_001013725.2), *Saccharomyces cerevisiae* ScGcn2 variants in which sequences were replaced by the corresponding human sequences as indicated (Gcn2‐HsA, Gcn2‐HsB, Gcn2‐HsC) and *S. cerevisiae* Gcn2 (ScGcn2, Accession: NP_010569.3) (Program ClustaW). Only the region of the alignment that encompasses the auto‐phosphorylation sites of HsGCN2 and ScGcn2 is shown (indicated as arrows at residues T899 and T904 for HsGCN2 and T882 and T887 for ScGcn2). Human residues are shown in red, and *S. cerevisiae* residues are shown in blue. Amino acids conserved between HsGCN2 and ScGcn2 are highlighted in black. (B) Yeast *gcn2Δ* strain H2557 was transformed with plasmids expressing the proteins as indicated (from top to bottom, plasmids pES806‐HsA‐p722, pES807‐HsB‐p722, pES808‐HsC‐p722, p722) or empty vector (pRS316). Transformants were grown to saturation, subjected to 10‐fold serial dilutions, and 5 μL of each dilution was transferred to solid medium alone (control) or containing 3AT at the indicated concentrations. Plates were incubated at 30 °C and the growth documented with a document scanner. Images were selected from time points at which growth differences were most clearly visible. At least four different biological replicates were analyzed and a representative result is shown. (C) Strains from (B) were grown to exponential phase, harvested, whole cell extracts generated, and equal amounts of aliquots subjected to sodium dodecyl sulfate‐polyacrylamide gel electrophoresis (SDS/PAGE) and western blotting using antibodies specific against ScGcn2, and Pgk1 as loading control. Two (Gcn2‐HsA and Gcn2‐HsB) or three (Gcn2‐HsC and ScGcn2) independent biological replicates were investigated, and a representative result is shown. (D) Western signals in (C) were quantified, the Gcn2 levels determined relative to that of Pgk1, and plotted relative to the Gcn2/Pgk1 ratio of the strain expressing ScGcn2. Error bars depict the standard error from at least two independent biological replicates. The values between the Gcn2 variants and ScGcn2 were not significantly different (Student's *t*‐test, *P* ≤ 0.05). Samples shown in the western blot were run on the same gel.

To enable easy detection of ScGcn2 auto‐phosphorylation using a commercially available antibody specific to phosphorylated HsGCN2, we progressively replaced increasingly larger segments surrounding the ScGcn2 auto‐phosphorylation site with the corresponding sequences from HsGCN2. We found that the variant with the shortest replacement—dubbed Gcn2‐HsC—was able to nearly fully restore the growth of a *gcn2Δ* strain under starvation conditions. When cells were subjected to starvation, Gcn2‐HsC was detectable by a commercially available human anti‐Gcn2‐P antibody. The Gcn2‐binding effector protein Gcn1 is essential for Gcn2 activation [16, 17, 18]. Consistent with this, as found for ScGcn2, Gcn2‐HsC auto‐phosphorylates and phosphorylates eIF2*α* in a Gcn1‐dependent manner. This supports the idea that the amino acid changes made in Gcn2‐HsC did not severely impact Gcn2 activation and function.

## Materials and methods

### Yeast strains and plasmids used

Yeast strains and plasmids used in this study are outlined in Tables 1 and 2. Plasmids for this study were constructed commercially (Genscript, USA).

**Table 1:** Strains used in this study.

Strain	Genotype	Source
H1511	*MATα ura3‐52 trp1‐63 leu2‐3112 GAL2* ^+^	[19]
H2557	Same as H1511 but *gcn2Δ*	[16]
ESY10069	Same as H1511 but *gcn1Δ gcn2Δ*	This work

**Table 2:** Plasmids used in this study.

Plasmid	Gene	Selectable marker	Vector or parent plasmid	Source
p722	*Gcn2*	*Amp* ^*R*^ *URA3*	pRS316, CEN6/ARSH4	[20]
pES806‐HsA‐p722	*Gcn2‐HsA* *	*Amp* ^*R*^ *URA3*	p722	This work
pES807‐HsB‐p722	*Gcn2‐HsB* *	*Amp* ^*R*^ *URA3*	p722	This work
pES808‐HsC‐p722	*Gcn2‐HsC* *	*Amp* ^*R*^ *URA3*	p722	This work
p1833	*Gcn1‐myc*	*Amp* ^*R*^ *LEU2*	pRS425, 2 μ	Marton & Hinnebusch
pRS316	*Vector*	*Amp* ^*R*^ *URA3*	vector, CEN6/ARSH4	[21]
pRS425	*Vector*	*Amp* ^*R*^ *LEU2*	vector, 2 μ	[21]
pHQ1093	gcn2::hisG::URA3::hisG disruption cassette, *Amp* ^*R*^			Qiu & Hinnebusch

* For ScGcn2 amino acids replaced by HsGCN2 amino acids see Fig. 1A.

The *gcn1Δ gcn2Δ* strain ESY10069 was generated by removing the *GCN1* and *GCN2* open reading frames (ORFs) in the strain H1511 as follows. *GCN1* was first knocked out following published procedures [22]. Briefly, a *GCN1* deletion construct was first produced via PCR using primers containing homology to regions flanking the *GCN1* ORF, following published instructions [22]. The PCR amplification yielded a DNA fragment comprising the *URA3* selectable marker flanked by loxP sites and sequences homologous to the regions upstream and downstream of *GCN1*. The resulting construct was introduced into strain H1511, where via homologous recombination the endogenous *GCN1* ORF was replaced by the *URA3* marker flanked by loxP sites. The *URA3* marker was subsequently excised by introducing into the cells a plasmid expressing the Cre recombinase, following published procedures [22]. The Cre recombinase containing plasmid was then evicted by growth of cells on YPD medium, plating for single colonies, and choosing a colony that upon testing was Ura and Leu auxotrophic. *GCN2* was deleted using the *Eco*RI‐ and *Xba*I‐digested plasmid pHQ1093 containing the *gcn2::hisG::URA3::hisG* disruption cassette, followed by the eviction of the *URA3* marker by growth on 5‐fluoroorotic acid medium.

### Semiquantitative growth assay

Semiquantitative growth assays were conducted following our previously described protocol [23]. In brief, saturated yeast cultures were serially diluted 10‐fold, and 5 μL of each dilution was transferred onto solid minimal medium. Plates were incubated at 30°C, and cell growth was monitored over a two‐week period using a document scanner.

### Generation of whole cell extracts for immunoblotting

Cell extracts were prepared as published previously [24]. In brief, saturated yeast cultures were used to inoculate liquid minimal medium and grown at 30 °C with shaking at 150 rpm until reaching exponential phase. At an OD (600 nm) of 0.6–0.7, 3AT was added to a final concentration of 15 mm, or no 3AT was added for the duration as indicated in the figure. Then, the cultures were transferred into a mixture of ice chips and formaldehyde (1% final concentration). After 1 h of incubation and mixing every 15 min, excess formaldehyde was neutralized by adding glycine. Cell pellets were generated, washed with ice‐cold double‐deionized water and stored at −80 °C. For chemical lysis, one‐tenth of the cell pellets were resuspended in 200 μL of 0.1 m NaOH. After a 5‐min incubation at room temperature, the cells were pelleted and the pellet was resuspended in 200 μL of 2× denaturing protein sample buffer (4% SDS, 20% glycerol, 120 mm Tris–HCl pH 6.8, 0.1% Bromophenol blue; with 10% (v/v) 2‐mercaptoethanol added just before use), and then incubated for 8 min at 80 °C.

### Immunoblotting

Equal volumes of lysed cell pellets were resolved through sodium dodecyl sulfate (SDS) denaturing polyacrylamide gel electrophoresis (SDS/PAGE) using 4–17% gradient gels. Gels were prepared using a 40% acrylamide/bisacrylamide (29 : 1 ratio) solution (Bio‐Rad, #1610146) following a protocol described previously [24, 25]. SeeBlue prestained protein standard was used as a size marker (LC5625; Thermo Fisher Scientific). Gels were run with 1× tris‐glycine SDS running buffer (1 610 732; Bio‐Rad) at 150 V until the dye front reached the bottom of the gel. Proteins were transferred to a nitrocellulose membrane (no.: 1620112; Bio‐Rad) using 1× tris‐glycine buffer (1 610 734; Bio‐Rad) containing 20% methanol at 100 V for 1 h 30 min. Membranes were probed with mouse monoclonal antibodies against Pgk1 (1 : 5000; no.: 459250; Pierce, Life Technologies), rabbit recombinant monoclonal antibodies against human Gcn2‐P (1:1000, no.: 75836; Abcam), and guinea pig antibodies against ScGcn2 (1 : 1000 [26]). The guinea pig antibodies were pretreated by adding whole cell extract of the *gcn2Δ* (H2557) strain (generated as described previously [27]) at a concentration of 1.5 mg per 10 mL, followed by overnight incubation at 4°C. The immune complexes were visualized using horseradish peroxidase‐conjugated donkey anti‐rabbit antibodies (1 : 100000, no.: 31458; Thermo Scientific), goat anti‐guinea pig IgG‐HRP antibodies (1 : 10000, sc‐2438; Santa Cruz Biotechnology) or goat anti‐mouse antibodies (1 : 50000, no.: 31430, Thermo Scientific). Detection was performed using the Pierce ECL Western Blotting Substrate (no.: 32106; Thermo Scientific, USA) and the ChemiDoc™ Imaging System (Bio‐Rad), and band intensities were determined with ImageJ software [28].

## Results & discussion

### Engineering ScGcn2 with human‐like auto‐phosphorylation site

To compare the amino acid composition surrounding the Gcn2 auto‐phosphorylation site, we performed a sequence alignment between HsGCN2 and ScGcn2 sequences. As seen in Fig. 1A, the sequences surrounding the auto‐phosphorylation sites are moderately conserved. Specifically, conserved residues are interspersed with residues of lower conservation. This may explain why the anti‐human GCN2 phospho‐Thr899 antibody cannot detect ScGcn2‐P (Fig. 1A). Their recognition likely depends not only on the phosphorylated residue itself but also on human‐specific amino acids flanking the auto‐phosphorylation site. For that reason, we aimed to modify the yeast sequences surrounding the auto‐phosphorylation sites to more closely resemble those of HsGCN2.

Anti‐human GCN2 phospho‐Thr899 antibodies are commercially available from various companies; however, none of them disclose the peptide sequence used to generate the antibodies. Therefore, in an effort to generate a ScGcn2‐P protein that can be detected by the anti‐human GCN2 phospho‐Thr899 antibody, three ScGcn2 variants were generated in which increasing areas flanking the auto‐phosphorylation site were replaced by the equivalent HsGCN2 sequence. In variant Gcn2‐HsA, a 52 amino acid segment (from K858 to Y909) was replaced by the corresponding human segment (from T871 to F927, 57 amino acids) (Fig. 1A). In variant Gcn2‐HsB, a 32 amino acid segment (from K858 to M889) was replaced with the equivalent human segment (from T871 to L906, 36 amino acids). Finally, in variant Gcn2‐HsC, a 12 amino acid segment (from L874 to I885) was replaced with the equivalent human segment (from K891 to V902, 12 amino acids). These plasmid‐borne variants were generated commercially from a plasmid expressing ScGcn2 from its native promotor (plasmid p722) [20].

### Gcn2‐HsC complements the 3‐amino‐1,2,4‐triazole sensitivity phenotype of a *gcn2Δ* strain

*In vivo*, Gcn2 activation can be easily scored by growth on solid medium containing 3‐amino‐1,2,4‐triazole (3AT), a drug eliciting histidine (His) starvation by inhibiting an enzyme in the His biosynthetic pathway [29]. In contrast to a wild‐type strain, a *gcn2Δ* strain is unable to grow in the presence of 3AT, due to its inability to overcome starvation. The plasmid‐borne Gcn2 variants, a plasmid bearing ScGcn2, and an empty vector, were each transformed into the *gcn2Δ* strain H2557. To evaluate the ability of the Gcn2 variants to reverse the 3AT‐induced growth defect of the *gcn2Δ* strain, the resulting transformants were subjected to semiquantitative growth assays, in which the amount of growth in the presence of 3AT serves as an indicator of the level of Gcn2 activation.

As expected, the *gcn2Δ* strain harboring vector alone was unable to grow in presence of 3AT, in contrast to the strain expressing ScGcn2 (Fig. 1B, row 7&8 vs 9). On medium lacking 3AT, all strains grew equally well, suggesting that the Gcn2 variants did not affect cell growth in general (Fig. 1B, left panel). In presence of 3AT, the cells expressing Gcn2‐HsA were hardly able to grow, and this 3AT sensitivity (3AT^s^) phenotype was comparable to that of the strain lacking Gcn2, suggesting that Gcn2‐HsA was not functional (Fig. 1B, row 1&2 vs 9). The cells expressing Gcn2‐HsB showed growth on medium containing low concentrations of 3AT, but with increasing 3AT concentration the cells were hardly able to grow, suggesting that Gcn2‐HsB is only partially functional (Fig. 1B, row 3&4 vs 7&8 vs 9). On the contrary, cells expressing Gcn2‐HsC were able to grow in presence of 3AT, almost as well as the control cells harboring ScGcn2 (Fig. 1B, row 5&6 vs 7&8 vs 9). Only at very high 3AT concentrations (150 mm) did cells expressing Gcn2‐HsC not grow as well as those expressing ScGcn2.

Western blotting revealed that the Gcn2 variants were expressed at least as well as ScGcn2 (Fig. 1C,D). This suggests that the inability of Gcn2‐HsA to complement a *gcn2Δ* strain was not due to insufficient expression levels.

Together, our findings suggest that Gcn2‐HsC, in which only a 12 amino acid segment is replaced with the corresponding human sequence, functions almost as well as ScGcn2.

### Gcn2‐HsC is capable of phosphorylating eIF2 *α*

**Fig. 2:**
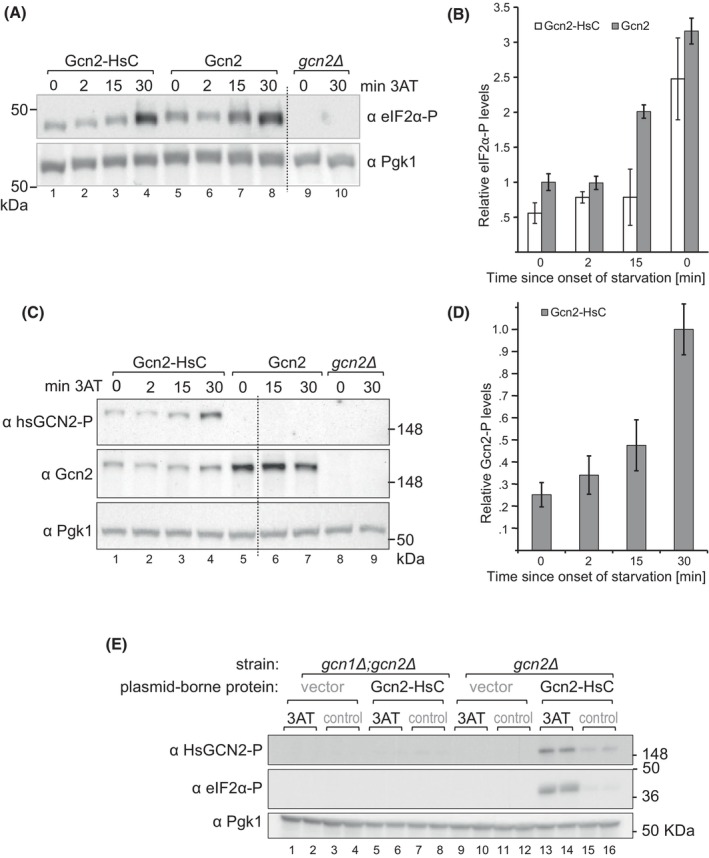
Gcn2‐HsC is capable of phosphorylating eIF2*α*, and of auto‐phosphorylation. (A) The *gcn2Δ* strain H2557 expressing the indicated proteins was grown to exponential phase, and then exposed to 15 mm 3AT for 0, 2, 15 or 30 min, as indicated. Cells were harvested, whole cell extracts generated and equal amounts of aliquots subjected to sodium dodecyl sulfate‐polyacrylamide gel electrophoresis (SDS/PAGE) and western blotting using antibodies specific against the phosphorylated form of eIF2*α*, and Pgk1 as loading control. A representative result is shown from four biological replicates investigated. Samples shown in the western blot were run on the same gel, the dotted line indicates the removal of lanes between lanes 8 and 9. (B) Western signals in (A) were quantified and the eIF2*α*‐P levels determined relative to that of Pgk1, and plotted relative to the eIF2*α*‐P/Pgk1 ratio of the nonstarved strain expressing ScGcn2. Error bars depict the standard error from four biological replicates. For each time point, the values between the ScGcn2 and Gcn2‐HsC strains were not significantly different (Student's *t*‐test, *P* ≤ 0.05). (C) Equal amounts of whole cell extract from (A) were subjected to SDS/PAGE and western blotting using anti‐human GCN2 phospho‐T899 antibodies, and anti‐Pgk1 as loading control. The membrane, previously probed with anti‐human GCN2 phospho‐T899 antibodies (top image), was subsequently probed with anti‐Gcn2 antibodies without prior removal of the anti‐human GCN2 phospho‐T899 antibodies (middle image), to ascertain that both antibodies detected bands at the same molecular weight. A sample result is shown from four biological replicates investigated. Samples shown in the western blot were run on the same gel, the dotted line indicates the removal of a lane between lanes 5 and 6. (D) Western blot signals in panel (C) were quantified, and the Gcn2‐P/Pgk1 ratio calculated for each sample. These values were then normalized to the Gcn2‐P/Pgk1 ratio of the 30‐min starved strain expressing Gcn2‐HsC, the resulting data plotted. Error bars depict the standard error from four biological replicates. (E) Two independent transformants of each *gcn1Δ gcn2Δ* strain ESY10069 and *gcn2Δ* strain H2557—expressing the proteins as indicated—were subjected to 3AT starvation for 30 min or not (control), harvested, extract generated and subjected to western blotting as done in (A) and (B). Samples shown in the western blot were run on the same gel.

In order to verify that Gcn2‐HsC is a fully functional kinase, we next scored for the phosphorylation levels of the Gcn2 substrate, eIF2*α*. *gcn2Δ* cells expressing Gcn2‐HsC, ScGcn2, and no Gcn2, respectively, were grown to exponential phase, after which 3AT was added at a final concentration of 15 mm. After 2, 15 or 30 min, the cultures were harvested, and the generated cell extracts subjected to western blotting using antibodies specific against phosphorylated eIF2*α* (eIF2*α*‐P), and against Pgk1 as control for equal loading. While in a *gcn2Δ* strain, no signal was detectable for eIF2*α*‐P, under unstarved conditions basal eIF2*α*‐P levels were detectable for cells expressing ScGcn2 or Gcn2‐HsC (Fig. 2A lane 9&10 vs 1 vs 5, Fig. 2B). With increasing duration of starvation, cells harboring Gcn2‐HsC showed increased levels of eIF2*α* phosphorylation, as found for cells expressing ScGcn2 (Fig. 2A lanes 1–4 vs 5–8, Fig. 2B). When quantifying the eIF2*α*‐P signals relative to the Pgk1 level for each sample, for each time point the eIF2*α*‐P level of the Gcn2‐HsC expressing strain appeared to be lower than that of the strain harboring ScGcn2, although the difference seemed to not be statistically significant. Taken together, our findings demonstrate that Gcn2‐HsC is capable of phosphorylating its substrate eIF2*α*.

### Anti‐human GCN2 phospho‐Thr899 antibodies detect activated Gcn2‐HsC

Gcn2 needs to auto‐phosphorylate before it can phosphorylate eIF2*α* [1]. To score for phospho‐Thr899 in Gcn2‐HsC, aliquots from the samples generated above for determining eIF2*α*‐P levels were subjected to western blotting using anti‐human GCN2 phospho‐Thr899 antibodies and antibodies against Pgk1. We found that the anti‐human GCN2 phospho‐Thr899 antibody detected a band at the expected molecular weight of Gcn2 (approximately 170 kDa), and the intensity of this band increased with the duration of starvation (Fig. 2C lanes 1–4, Fig. 2D). No band could be detected in samples containing ScGcn2 even after prolonged starvation, in agreement with previous reports that the anti‐human GCN2 phospho‐Thr899 antibody is unable to detect the phosphorylated form of ScGcn2 (Fig. 2C, lanes 5–7).

Under nonstarvation conditions, the anti‐human GCN2 phospho‐Thr899 antibody weakly detected Gcn2‐HsC (Fig. 2C, lane 1), perhaps reflecting modest activation of GCN2 under these conditions, consistent with the low levels of eIF2*α*‐P observed in the absence of 3AT (Fig. 2A), or cross reaction of the antibody with unphosphorylated Gcn2‐HsC. In order to test whether the anti‐human GCN2 phospho‐Thr899 antibody detects unphosphorylated Gcn2‐HsC, we took advantage of the fact that Gcn2 absolutely requires Gcn1 for its activation [17]. Plasmid‐borne Gcn2‐HsC and vector alone, respectively, were introduced into isogenic *gcn1Δ gcn2Δ* strain. Then, the transformants were exposed to starvation in liquid medium by the addition of 3AT, and the generated whole cell extracts subjected to western blotting, as done above. We found that the anti‐human GCN2 phospho‐Thr899 antibody detected a signal when the Gcn2‐HsC harboring strain also contained Gcn1, while no detectable signal was observed in the absence of Gcn1 (Fig. 2E, lanes 5&6 vs 13&14). This supports the idea that the anti‐human GCN2 phospho‐Thr899 antibody used in this study was specific to the phosphorylated form of Gcn2‐HsC.

Together, our findings suggest that the anti‐human GCN2 phospho‐Thr899 antibody is able to specifically detect the phosphorylated form of Gcn2‐HsC.

### Gcn2‐HsC is dependent on Gcn1 for function

**Fig. 3:**
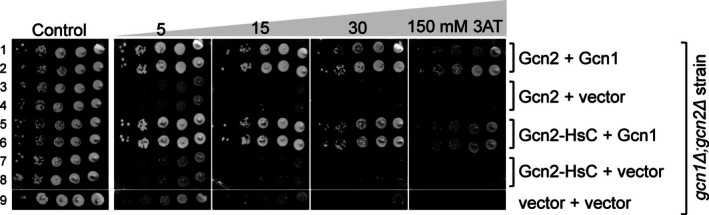
Gcn2‐HsC function is dependent on Gcn1. The *gcn1Δ* g*cn2Δ* strain ESY10069 was transformed with two plasmids expressing the proteins as indicated (plasmid set 1 expressing Gcn2 variants or not: from top to bottom p722, pES808‐HsC‐p722, or empty vector pRS316; plasmid set 2 expressing Gcn1 or not: p1833 or empty vector pRS425). A semiquantitative growth assay was performed as done in Fig. 1B. A representative image is shown from at least 4 biological replicates.

Gcn2 absolutely requires its effector protein Gcn1 to be able to detect starvation and become activated [17]. To confirm that this is still true for Gcn2‐HsC, semiquantitative growth assays were conducted using a *gcn1Δ gcn2Δ* strain transformed with two plasmids. One plasmid expressed Gcn2‐HsC, ScGcn2, or no protein (empty vector), respectively, and the other expressed either Gcn1 or no protein (empty vector). Subsequent semiquantitative growth assays revealed that all transformants grew equally well on the control plate, as expected (Fig. 3). We found that cells expressing ScGcn2 or Gcn2‐HsC were able to grow on medium containing 3AT, but only when Gcn1 was also present (Fig. 3, rows 1&2 vs 3&4, 5&6 vs 7&8). In the absence of Gcn1, cells showed severely hampered growth in the presence of 3AT. This strongly suggests that Gcn2‐HsC depends on Gcn1 to mount an effective starvation response, supporting the notion that, like native ScGcn2, Gcn2‐HsC requires Gcn1 for its activation.

## Conclusion

The straightforward detection of auto‐phosphorylated ScGcn2 has remained challenging due to the lack of potent phospho‐specific antibodies. Here, we engineered the auto‐phosphorylation site of ScGcn2 to resemble that of the human protein, enabling detection of auto‐phosphorylation by a commercially available anti‐human GCN2 phospho‐Thr899 antibody. Gcn2‐HsC almost completely complemented a *gcn2Δ* strain. The minor incompleteness in complementation may reflect subtle impairment in activation, phosphorylation efficiency, or in binding eIF2*α* or other molecules essential for activation. Nevertheless, since incomplete complementation was observed only at extremely high 3AT concentrations, our results suggest that Gcn2‐HsC serves as a valuable tool for Gcn2‐related studies in the highly tractable yeast system. Additionally, direct comparisons between Gcn2‐HsC, ScGcn2, and HsGCN2 could help enhance our detailed understanding of the molecular basis of Gcn2/GCN2 auto‐phosphorylation.

## Conflict of interest

The authors declare no conflicts of interest.

## Author contributions

The idea was conceived and the constructs designed by ES, experiments conducted by RAA and AHS, results interpreted by all, manuscript written by ES with contributions from AAS and RAA.

## Data Availability

The data that support the findings of this study are available in the figures and tables of this article.
